# THE CONTRIBUTION OF THE MICROBIOME TO THE AGING PROCESS

**DOI:** 10.1093/geroni/igae098.1911

**Published:** 2024-12-31

**Authors:** Dario Riccardo Valenzano

**Affiliations:** Leibniz Institute on Aging, Jena, Thuringen, Germany

## Abstract

The human microbiome, a complex ecosystem of microorganisms residing in our bodies, is emerging as a critical contributor to our overall health and the aging process. With a total number of genes that vastly outnumbers our own—approximately 100 to 1—the microbiome influences virtually all biological processes, from nutrient absorption and immune system development to protection against pathogens. This diverse and dynamic community, acting as a polypharmacy, produces a plethora of compounds that affect our metabolism and are linked to various health outcomes. Recent findings have highlighted that numerous diseases, including metabolic disorders, immune-related conditions, and even neurodegenerative diseases are associated with the disruptions of the microbiome-host balance. Given its extensive involvement in health and disease, the microbiome has become highly relevant in aging research. Scientists are now investigating how changes in the microbiome composition throughout our lives can lead to new anti-aging interventions. One of the most promising areas of research is microbiome transplantations, which offer a novel strategy to potentially slow down or reverse aspects of the aging process by modifying our internal microbial landscape. Moreover, the discovery that rapamycin, a powerful anti-aging drug, originates from microbes underscores the microbiome’s potential as a treasure trove for developing new anti-aging compounds. By understanding and harnessing our microbiome, we stand on the brink of revolutionizing anti-aging strategies, transforming our approach to disease prevention in our later years. This rapidly expanding research field promises to unlock new possibilities for enhancing longevity and improving the quality of life as we age.

